# Beneficial medicinal effects and material applications of rose

**DOI:** 10.1016/j.heliyon.2023.e23530

**Published:** 2023-12-10

**Authors:** Hsiuying Wang

**Affiliations:** Institute of Statistics, National Yang Ming Chiao Tung University, Hsinchu, 300093, Taiwan

**Keywords:** Rose, Biomaterial, Medical effect, Nano, Pharmaceuticals, engineering

## Abstract

Rose is a beautiful and fragrant plant with a variety of medicinal and substance uses. Various parts of rose such as fruits, flowers, leaves, and bark can be used in various product development, including cosmetics, food, pharmaceuticals, and engineering. The medical benefits of roses include the treatment of inflammation, diabetes, dysmenorrhea, depression, stress, seizures, and aging. Rose water is precious beauty water for skin care and has antibacterial effects on various microbiota. The surface of a rose petal exhibits a hierarchical structure comprising microscale papillae, with each papilla further featuring intricate nanofolds. With this structural feature, rose petals have high water contact angles together with antagonistic wetting properties. The hierarchical structures of rose petals were shown to have anti-reflection and light-harvesting abilities, which have the potential to be materials for various electronic products. Rose petals are an excellent biomimetic/bioinspired material that can be applied to the popular material graphene. This paper reviews the medical function and material application of roses. During the COVID-19 pandemic, medical materials or food shortages have become a global issue. Natural biomaterials could be a good alternative. Roses, with so many benefits, definitely deserve more exploration and promotion.

## 1 Introduction

**Fig. 1:**
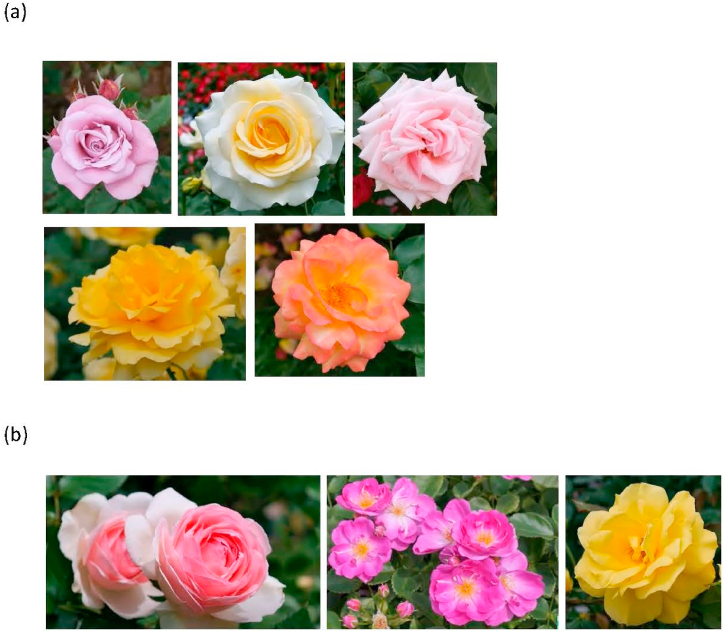
Images of different types of roses: (a) Rosa hybrid; (b) other types.

The rose, derived from the Latin word Rosa and belonging to the Rosaceae family, is classified as a flowering shrub (Fig. 1 (a)-(b)). The Rosa extract can be used to prevent or treat various disorders. There are over 100 different species of Rosa genus that are extensively distributed throughout Europe, the Middle East, Asia, and North America. The common petal in eastern Asia, Rosa rugosa has been traditionally used as an herbal medicine for ailments such as stomach aches, diarrhea, menoxenia, diabetes mellitus, pain, and chronic inflammatory diseases [1]. Rosa rugosa can be found across various temperate regions of eastern Asia, encompassing countries such as China, Japan, and Korea. Rosa canina is the most abundant and studied Rosa species in Europe that contains high concentrations of vitamin C, quercetin, and ellagic acid [2]. Rosa damascena Mill., known as the Damask Rose, is renowned for its fine fragrance, and it is a source of rose oil for use in perfumery to make rose water. As the king of flowers, Rosa damascene has long symbolized love, purity, faith, and beauty throughout history.

Various components of medicinal plants, such as roses, including fruits, flowers, leaves, roots, and bark, can be used for medicinal purposes. Different plant parts have different healing properties. Throughout ancient times, certain flowers have been employed in the treatment of numerous diseases [3]. Calendula officinalis is an annual or perennial herb in the Asteraceae family, native to southern Europe and the Eastern Mediterranean area, which has been widely used for medicinal, dye, and cosmetic purposes. Over the past decade, Calendula officinalis has played a significant role in wound care and has been historically utilized for its benefits in treating jaundice, purifying the blood, and acting as an antispasmodic [4]. Vision loss and the death of retinal cells can be attributed to retinal degenerative diseases. To assess the protective effects on retinal function, an animal study was carried out, examining a complex lutein formula that combined several natural compounds, including Calendula officinalis, Lycium barbarum, Vaccinium myrtillus, Cassia obtusifolia, and Rhodiola rosea [5]. The results demonstrated that supplementation with this complex lutein formula was effective in reducing the loss of retinal function caused by prolonged exposure to light. St. John's wort, named after John the Baptist, is a plant that grows in the wild, has been used for centuries for treating depression in Europe. In individuals experiencing mild-to-moderate depression, St. John's wort exhibited similar effectiveness and safety as selective serotonin reuptake inhibitors, commonly prescribed antidepressant medications [6]. Lilies, which are bulbous herbaceous plants, are characterized by their striking large flowers. These plants possess therapeutic potential for treating a range of conditions, particularly as anti-inflammatory and antioxidant agents [7].

In addition to their use in medical therapy, many plants have industrial material applications. Dye-sensitized solar cells (DSSCs) offer cost-effective solar energy options with notable advantages. The light-absorbing effectiveness of Lily (Iris Persica) dyes and the synthesis of ${TiO}_{2}$ nanofibers were examined [8]. DSSCs with higher efficiency resulted from improved light absorption and pigment-nanofiber adsorption. Canna lily pigments were used for eco-friendly solar cells, achieving high charge efficiency on ${TiO}_{2}$ photoanode, and fine-tuning performance parameters via impedance spectroscopy [9]. Like other plants, roses have the potential to be utilized as materials in various applications.

Synthetic and bio/natural polymers are two types of polymers. Bio/Natural polymers occurring in nature were either chemically synthesized from biological materials or entirely biosynthesized by living organisms. Traditional synthetic polymers are difficult to recycle and can remain in nature for a long time. The utilization of biopolymers as a sustainable substitute for synthetic polymers has been proposed to reduce the amount of landfill waste and the associated environmental burden [10]. Biopolymers derived from biomass, including polysaccharides, proteins, and lipids, obtained through the fermentation of yeast biomass, algae, or bacteria, have proven to be valuable resources for medical devices, food packaging, agricultural films, film processing applications, and sustainable clothing [11].

Due to resource constraints, especially from fossil fuels, the development of plants for the sustainable synthesis of materials has been promoted [12]. The rose is a valuable plant with the potential for various material applications. The hierarchical structure of rose petals, composed of microscale papillae and intricate nanofolds, confers unique properties such as high water contact angles and antagonistic wetting behaviors. Beyond these characteristics, rose petals also exhibit anti-reflection and light-harvesting abilities, making them potential candidates for electronic applications. Their biomimetic potential extends to materials like graphene, offering opportunities for enhancing electronic devices through their distinctive design. This makes rose petals a valuable resource in the pursuit of advanced materials.

In this paper, the medical effects and polymer application of roses are reviewed and discussed.

## 2 Structure and compounds of roses

**Fig. 2:**
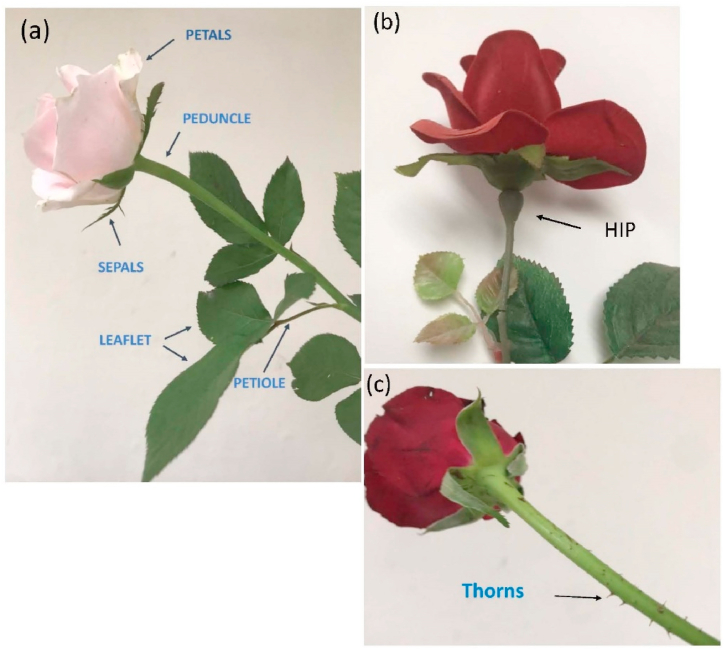
(a) Petals, peduncle, sepals, leaflets, and petioles of rose; (b) hip of rose; (c) thorns of rose.

The structure of a rose can be divided into several key parts, which include petals, roots, stems, leaves, buds, flowers (sepals, stamens, pistils), thorns, and hips. Fig. 2 (a)-(c) plots some of these structural elements.

Different parts of roses have different chemical compositions. The type and percentage of chemical components depend on the variety of rose, growing environment, harvest time, and processing method. Some important components of different rose parts for industry or medical applications are listed in Table 1.

**Table 1:** Important components of three popular used parts of roses for industry or medical applications.

Parts	Chemical compound	Possible industry or medical applications
Petal	terpenes, aroma alcohols, flavonoids, anthocyanin, phenolic acids, polyphenols, aldehydes, ketones, tannins, vitamin C (ascorbic acid), carotenoids, minerals	analgesic, anticonvulsive, hypnotic, cardiovascular, laxative, and antioxidant properties, aromatherapy, perfumery, cosmetics and skincare, teas, natural colorant
Hips	vitamin C (ascorbic acid), carotenoids, flavonoids, polyphenols, tannins, pectin, vitamin E (tocopherol), fatty acids, phytosterols	anti-Inflammatory and antioxidant properties, dietary supplements, cosmetics and skincare, natural coloring
Leaves	flavonoids, phenols, hydroxycinnamic acids, tannins, terpenes, aldehydes, alcohols, vitamin C, fatty acids, minerals.	diuretic properties, natural dyes, mild astringent properties

The Rosa damascene flowers contain various components, including flavonoids, terpenes, polyphenols, anthocyanin, and glycosides. The volatile oil extracted from Rosa damascene flowers encompasses analgesic, anticonvulsive, hypnotic, cardiovascular, laxative, and antioxidant properties and has been utilized as a raw material in the perfumery, cosmetics, and pharmaceutical industries [13,14]. The rose petals also contain aroma compounds such as aroma alcohols, phenolic compounds, and aldehydes, which contribute to the unique fragrance of rose [15].

Rose hips are rich in vitamins, notably vitamin C, alongside phenolic compounds, carotenoids, tocopherol, bioflavonoids, tannins, volatile oils, and pectins [16,17]. The nutritional quality of Sicilian rose hips is substantial, making them appropriate for utilization as functional foods due to their unique biochemical composition and their potential as promising reservoirs of natural antioxidants [18]. Leaves of Rosaceae could be sources of flavonoids, and the polyphenolic compounds as well as the bioactivity of leaf extracts from the Rosaceae family were investigated [19]. Leaf extracts of Rosa canina and Rosa rubiginosa had high total phenolic contents.

Bioactive phenolic compounds are powerful antioxidants used in traditional medicine. These nutritional elements contribute to the popularity of roses as ingredients in various dishes [20]. Other edible flowers include chrysanthemum [21], hibiscus [22], lavender [23], pansy [24], nasturtium [25,26], osmanthus, and so on. They have antioxidant, antiproliferative, and antibacterial potential and their phenolic compounds were responsible for the bioactivities [27]. Edible flowers offer a novel potential as a natural source of antioxidants, providing oxidative protection for cold-pressed oils that are abundant in omega-3 fatty acids [28]. Having a variety of nutritional and bioactive components, edible flowers have become a popular diet trend [29,30]. Among these edible flowers, the benefits of the rose may outweigh the others [31].

## 3 Effect of treatment

Healing with medicinal plants has a long history. Since ancient times, people looked for drugs in nature to treat various diseases [32]. Medicinal plants are the result of trial and error by humans for hundreds of centuries, in search of food that can be used for the treatment of diseases [33]. Herbal medicines were natural and have been shown to have good effects in controlling infectious diseases. The coronavirus disease 2019 (COVID-19), which resulted in a global pandemic, was caused by the severe acute respiratory syndrome coronavirus 2 (SARS-CoV-2) [[34], [35], [36]]. Promising outcomes were observed in the treatment of COVID-19 through the combined approach of herbal medicine and Western medicine [37]. Other natural ingredients such as collagen can also be used to heal certain comorbidity of COVID-19 with fewer side effects than chemical drugs [38]. In addition, medicinal plants were shown to have good effects in the treatment of cardiovascular disease, Parkinson's disease, and cancer [[39], [40], [41]]. In this section, the treatment effects of Rosa aromatherapy and Rosa extract for various diseases are reviewed.

### 3.1 Rosa aromatherapy

#### 3.1.1 Dysmenorrhea treatment

Dysmenorrhea is one of the most common gynecological problems for women. The prevalence rate of dysmenorrhea varied in different countries that might be ranged from 28 % to 78 % [42,43]. Women with dysmenorrhoea might have difficulties with vaginismus and chronic pain syndromes [44]. Various medications have been employed to alleviate menstrual pain, including anti-inflammatory drugs, oral contraceptives, and analgesics. However, prolonged usage of these medications may lead to side effects like nausea, dyspepsia, peptic ulcers, and diarrhea [45]. A randomized clinical trial study aimed to investigate the effects of Rosa damascene essential oil administered by inhalation on patients with primary dysmenorrhea [46]. The study encompassed a group of 100 patients, aged between 19 and 30 years, who were admitted to the emergency unit and diagnosed with primary dysmenorrhea. A control group was administered diclofenac sodium (75 mg/IM) alone, while the experimental group received diclofenac sodium along with aromatherapy using 2 % rose essential oil. The aromatherapy group exhibited a significant outcome when compared to the control group. This revealed that the nonpharmacologic aromatherapy as an adjuvant to conventional treatment methods could be beneficial for pain relief in women with primary dysmenorrhea.

In addition to investigating the rose oil effect by inhalation, the effect of massage therapy with rose oil was studied. In a randomized controlled trial, a total of 75 participants were divided into three groups: (1) a massage group using rose oil, where participants conducted self-massage with Rose damascene, (2) a placebo group that performed self-massage with unscented almond oil, and (3) a no-treatment control group that solely performed self-massage [47]. In the next two cycles, all three groups received the intervention on the first day of menstruation. In the second cycle, the first group using massage therapy along with rose oil showed a significant reduction in dysmenorrhea pain in comparison with the other two groups. Another randomized blind clinical trial of crossover design was conducted to investigate the effect of the essential oils (cinnamon, clove, rose, and lavender in a base of almond oil) on menstrual pain [48]. In the initial treatment phase, participants in the first group received a daily aromatherapy abdominal massage using essential oils for 7 days before menstruation, while those in the second group underwent a similar intervention but with a placebo oil (almond oil). In the subsequent treatment phase, the two groups switched to the alternate regimen. The intensity and duration of menstrual pain and the amount of menstrual bleeding were significantly lower in the aromatherapy group than in the placebo group during both treatment periods. As a result, aromatherapy can be used as a nonpharmacological pain management to women suffering of dysmenorrhea, or excessive menstrual bleeding.

#### 3.1.2 Depression, stress, and anxiolytic treatment

Major depressive disorder (MDD) is a prevalent and severe mood disorder characterized by symptoms that can include anxiety, disturbances in sleep patterns, and diminished interest in usual activities. The two anxiety-related diseases gastroesophageal reflux and migraine both are associated with MDD [49,50]. The side effects of anti-anxiety medications include heart rhythm dysfunction, sudden death, and drug dependency. Rosa aromatherapy can be a complementary treatment along with routine pharmaceutical therapies to reduce anxiety and increase sleep quality in anxiety patients. The effects of anti-depressant, psychological relaxation, improving sexual dysfunction, and anti-anxiety effects were reported for rose oil [51].

A randomized clinical trial was conducted to investigate the effect of Rosa damascene fragrance on the anxiety and sleep quality of hospitalized patients in the cardiac care unit [52]. The result showed that the use of Rosa damascene in aromatherapy had a positive impact on anxiety reduction and enhanced the sleep quality of patients. The effects of Rosa damascena on autonomic parameters such as blood pressure, breathing rate, blood oxygen saturation, pulse rate, and skin temperature, as well as on emotional responses related to relaxation, vigor, calmness, attentiveness, mood, and alertness were investigated [53]. When compared to a placebo, the application of rose oil resulted in noteworthy reductions in breathing rate, blood oxygen saturation, and systolic blood pressure. Participants in the rose oil group also reported feeling more serene, relaxed, and less alert compared to those in the control group. These results indicate that rose oil has a relaxing effect.

Selective serotonin reuptake inhibitors (SSRIs) are the most commonly prescribed antidepressants. However, treating MDD with SSRIs may have a negative effect on sexual function. Adjuvant Rosa damascena oil could improve MDD and SSRI-induced sexual dysfunction in male patients, but the effect on female patients is not significant [54]. A double-blind and randomized clinical trial for a total of 60 male patients suffering from MDD and SSRI-induced sexual dysfunction were randomly assigned to the Rosa damascena oil group or a placebo group [54]. The results showed that the use of Rosa damascena oil in male patients with both MDD and sexual dysfunction induced by SSRIs demonstrated an improvement in sexual dysfunction.

Stress is a reaction to mental or emotional pressure that may disturb the psychological or physiological homeostasis of the body. Anti-stress medications may have the cost of severe adverse effects. An animal study was conducted to evaluate the effect of Rosa moschata extract in alleviating stress using the acute restraint model in mice [55]. The findings showed that Rosa moschata could significantly alleviate stress.

In addition, the effect of Rosa damascene aromatherapy on state anxiety and sleep quality among a population of Iranian operating room (OR) personnel during the COVID-19 pandemic was evaluated [56]. Eighty OR personnel were divided into two groups using the stratified randomization method: Rosa damascene group and a placebo (paraffin oil) group. The result showed that Rosa damascene aromatherapy can be effective in reducing state anxiety and improving the sleep quality of OR personnel.

### 3.2 Rose extracts for therapeutic uses

#### 3.2.1 Anti-inflammation

In response to various detrimental stimuli like infection, chemical exposure, tissue damage trauma, or exposure to bacterial components, inflammation acts as a protective mechanism Macrophages are innate immune cells that can produce inflammatory molecules to respond to microbial threats, eliminate pathogens and promote tissue repair. Dysfunctional macrophage responses, such as macrophage activation syndrome caused by severe infections, including SARS-CoV infection, can be damaging to the host [57]. The anti-inflammatory effect of Rosa rugosa flower extract on lipopolysaccharide-stimulated RAW264.7 macrophages was demonstrated [58]. For the prevention and treatment of coronary artery disease, the clinical use of Rosa rugosa preparation has been established for a significant period [59]. Rosa rugosa flavonoids (RRF) extracted from petals of Xinjiang Sprig Rosa rugosa could alleviate acute myocardial ischemia-reperfusion injury (MIRI) in isolated rats’ hearts [60]. In a mouse MIRI model, the protective effects and underlying mechanisms of RRF were compared to those of compound danshen dropping pills [61]. The result showed that RRF significantly inhibited MIRI through anti-oxidative, anti-inflammatory, and anti-apoptosis effects.

Neuroinflammation is associated with the pathogenesis of neurodegenerative disorders. Flavonoids can modulate the inflammatory response and have neuroprotective effects. The flavonoid-rich extract obtained from Rosa laevigata Michx fruit had neuroprotective effects against cerebral ischemia-reperfusion that induced injury in rat brains [62]. The fruits of Rosa canina exhibited notable antinociceptive and anti-inflammatory properties [63].

#### 3.2.2 Anti-diabetic effect

Diabetes mellitus is an endocrinological disorder that is due to either the pancreas not producing enough insulin or the body does not respond appropriately to insulin [64]. There are several types of diabetes. In type 1 DM, the pancreas does not generate enough insulin to maintain normal levels of glucose in the blood. Digestive enzymes such as alpha-glucosidase are suggested as an herbal remedy for diabetes. The Rose damascena extract showed more than 50 % inhibitory effect on alpha-glucosidase [65]. Oral administration of a Rose damascena extract significantly decreased blood glucose in a rat animal study [66,67]. These findings suggested that Rosa damascena might exert an anti-diabetic effect by suppressing carbohydrate absorption from the intestine and could reduce the postprandial glucose level.

The protective effect of Rosa damascena essential oil on diabetes-induced testicular damage in rats was studied [68]. In comparison to the untreated group, rats that received rose oil treatment, particularly at higher dosages, exhibited higher sperm counts and increased diameters of seminiferous tubules. Moreover, rose oil was found to significantly elevate the cell count of spermatogonia, primary spermatocytes, Sertoli cells, and Leydig cells.

Rose pomace is a by-product of the essential oil extraction process that is rich in dietary fiber. Insoluble dietary fiber (IDF) has a better anti-obesity effect than soluble dietary fiber that can increase insulin sensitivity to reduce the risk of type 2 diabetes. IDF can be extracted from rose pomace [69]. Water-soluble polysaccharides extracted from Rosa roxburghii Tratt fruit by hot water method could potentially be an inhibitor of alpha-glucosidase [70]. The antidiabetic and antioxidant properties of the chemical composition of Rosa rugosa Thunb. were evaluated by analyzing spectral effect relationships [71]. The results showed that contributed the most to antioxidant and antidiabetic effects.

#### 3.2.3 Seizure

A seizure is a sudden and uncontrolled electrical activity in the brain that causes temporary abnormalities in muscle tone, movements, behaviors, sensations, or states of consciousness. Epilepsy is one of the most common chronic neurological disorders characterized by repeatedly occurring epileptic seizures. Oxidative damage plays an important role in the causation of several central nervous system malfunctions. Oxidative stress and inflammation are involved in seizure-related neurotoxicity. A Rosa hybrida petal extract was shown to have a neuroprotective effect in mice by examining their behavioral epileptiform seizures, biochemical, and morphological parameters of oxidative stress and inflammation [72].

In a rat model study, the hydroalcoholic extract of Rosa damascena significantly prolonged seizure latency and decreased the frequency and amplitude of pentylenetetrazole (PTZ) injection-induced epileptiform burst discharges [73]. Another rat model study explored the probable effects of Rosa damascena on neuronal apoptosis in the hippocampus of PTZ-induced seizures [74]. The finding revealed that the hydro-alcoholic extract of Rosa damascena exerts neuroprotective effects of seizure through a significant reduction of apoptotic neurons in several subregions of the hippocampal formation.

#### 3.2.4 Antimicrobial effects

The antimicrobial effects of rose water on various microbiota have been discussed in the literature. The antioxidant and antimicrobial properties in freeze-dried extracts of Rosa rugose fruits and the effect of a selected extract on bacterial survival in model fluids imitating protein food were investigated [75]. The studied bacteria include Bacillus cereus, Staphylococcus epidemidis, Listeria innocua, Staphylococcus aureus, Enterococcus faecalis, Klebsiella pneumoniae, Pseudomonas aeruginosa, Proteus mirabilis, Escherichia coli, Salmonella enteritidis. The rose extract was found to strongly inhibit the growth of some of these bacteria, but not all. Extracts obtained from rose hips possess significant value as raw materials due to their potential antimicrobial activity. The effects of whole pseudo-fruit and flesh extracts of three Rosa sp. varieties against Staphylococcus spp. bacteria isolated as food contaminants were investigated [76]. Several strains of bacteria from the genus Staphylococcus were considered in the study including Staphylococcus epidermidis, Staphylococcus xylosus, Staphylococcus haemolyticus, Staphylococcus capitis, and Staphylococcus warneri. Rosa rugosa fruit extract showed the strongest antimicrobial properties among the studied extracts.

Rosa gallica var. aegyptiaca is a species belonging to the Rosaceae family. The antioxidant and antimicrobial potential of five extracts from Rosa gallica var. aegyptiaca leaves against Listeria monocytogenes, Bacillus subtilis, Staphylococcus aureus, Escherichia coli, Salmonella enteritidis, and Candida albicans was examined [77]. In the study, five different types of extracts, namely n-hexane, chloroform, methanol, methanol/water 80 %, and water, were utilized. Among these extracts derived from the leaves, the 80 % hydromethanol extract exhibited the highest extraction yield and total phenolic content. Moreover, it displayed remarkable antioxidant and antimicrobial activities against all the microbial strains tested. The antioxidant activities and antimicrobial effects of Rosa rugosa Thunb. var. plena Regal flower cell sap (RFCS) were investigated [78]. RFCS was shown to have antioxidant activities and antimicrobial effects against many bacteria. Rosa damascena was rich in biologically active compounds of high value in the food, pharmaceutical, and cosmetic industries. Some selected rose damascena essential oil could act as a potentially promising strategy for fighting microbial strains [79].

#### 3.2.5 Anti-aging and skin repair

Aging is a major cause of many degenerative diseases, and the most intuitive consequence of aging is manifested in the skin [80]. The process of aging skin is influenced not solely by internal factors but also expedited by numerous external environmental elements, particularly ultraviolet (UV) radiation. A study showed that a mixture of extracts of Kochia scoparia and Rosa multiflora can be used as a therapeutic agent for photoaged skin with few epidermal side effects when applied twice daily on the dorsal skin of photoaged mice for 8 weeks [81]. Rosa gallica downregulated ultraviolet B (UVB)-induced cyclooxygenase 2 and matrix metalloproteinases-1 expression in the skin, and oral administration of Rosa gallica could prevent UVB-induced skin aging through targeting the c−Raf signaling axis [82]. The anti-aging and antioxidant properties of a 50 % ethanol-water extract derived from Rosa gallica petals exhibited notable inhibitory effects on tyrosinase activity, melanogenesis, and solar ultraviolet (UV)-induced matrix metalloproteinase 1 [83]. The 50 % EtOH extraction was optimal for the highest anti-aging, and anti-oxidative effects as well as to obtain the highest flavonoid content. The cosmetic anti-aging potential of the extract of Rosa centifolia stem was discussed. A hydroalcoholic extract of Rosa centifolia showed particular promise for its valuable anti-hyaluronidase and antioxidant activities, and its anti-elastase and anti-inflammatory potential [84]. The effect of extracts from Rosa gallica petals on skin whitening and anti-wrinkle activity was investigated [85]. The findings indicated that Rosa gallica petals could evoke skin whitening and anti-wrinkle formation activity by regulating intracellular signaling.

The development of Rosa floribunda charisma, a modern rose variety, occurred through the crossbreeding of tea roses and polyantha roses [86]. The utilization of synthesized nanoparticles derived from Rosa floribunda charisma holds great potential as a significant natural reservoir of antibacterial and anti-aging agents within the skin care cosmetic industry [87]. An enriched polyphenolic extract obtained from the by-product of Rosa damascena could delay age-related morbidities and extend flies’ lifespan [88]. Rosa gallica petals on skin aging-related activities such as skin whitening and anti-wrinkle properties were investigated. Among 16 herbal extracts used for topical application in cosmeceutical products, Rosa damascus extract was one of three herbal extracts with the most significant antioxidant activity and promising whitening effect with moderate anti-tyrosinase activity [89]. Rosa rugosa aqueous polyphenol (RAP) is a polyphenol in Rosa rugosa flower tea. Antiaging activities of RAP were investigated in the model organism Caenorhabditis elegans [90]. The results indicated that Rosa rugosa tea had health benefits and RAP had the potential to be developed as an anti-aging bioactive product.

Plant oils are known for their effects on the restoration of skin homeostasis including olive oil, oat oil, pomegranate seed oil, almond oil, and rose hip oil [[91], [92], [93], [94], [95], [96]]. Especially rose oil is used in various cosmetics. Despite the wide use of plant oils, allergic contact dermatitis has been reported. A patient was diagnosed with allergic contact dermatitis caused by Rosa mosqueta oil [97]. As a result, it should be careful of any allergic reactions when using these plant oils.

#### 3.2.6 Other effects

Some other effects of rose extract treatment on neurological diseases, gastrointestinal digestion, and liver disease have also been discussed in the literature. A methanolic extract of Rosa x hybrida was evaluated for its antiproliferative properties in ovarian carcinoma cells [98]. The extract could promote antiproliferative activity in ovarian carcinoma cells by inducing autophagy and apoptosis. In addition, this Rosa x hybrida extract could be also used to develop new treatments for Alzheimer's disease.

The effects of simulated gastrointestinal digestion on rose and nasturtium flower extracts were evaluated, and flavonoids and phenolic acids were found to be the major classes of phenolic compounds detected in rose and nasturtium [99]. As roses and nasturtiums exhibited some phenolic compounds with high bioaccessibility and retained some bioactivity properties after simulating gastrointestinal digestion, they were recommended for human consumption.

Excessive alcohol drinking is a critical risk factor for liver dysfunction. Hepatocyte apoptosis is involved in the pathogenesis of alcohol-related liver disease (ALD), so anti-apoptotic extracts are of great importance in the prevention or treatment of ALD. Rosa rugosa extracts were shown to prevent EtOH-induced apoptosis in HepG2 cells via the activation of the AMPK/SIRT1 signaling pathway, revealing the anti-alcohol and hepatoprotective benefits of consuming rose [100]. The nutrition of the Rosa rugosa flower including vitamin C, flavonoids, and anthocyanins could be well retained by infrared freeze drying [101].

Migraine headache is a common neurological disorder. In traditional Persian medicine, Rosa damascena oil has been traditionally employed for treating migraines. To assess its impact on migraines, a randomized double-blinded placebo-controlled crossover trial was conducted involving 40 patients, utilizing a topical formulation of Rosa damascena oil [102,103]. The results showed that syndrome differentiation might help select patients who may benefit from the topical Rosa damascena oil for short-term relief of migraine pain. Alzheimer's disease is a neurodegenerative disease. A study showed that Rosa damascena could reverse behavioral deficits in a rat model of amyloid-β-induced Alzheimer's disease, suggesting that Rosa damascena might provide a new potential option for the prevention and treatment of Alzheimer's disease [104].

The pharmacological and clinical effects of Rosa canina were evaluated [105]. Rosa damascene is prescribed for chest and abdominal pain, constipation, digestive disorders, menstrual bleeding, and liver diseases [106]. The anti-HIV, anti-microbial, anti-infection, bronchodilatory, antitussive, cardiotonic, cardioaccelerating, and hypoglycemic effects of Rosa damascena have been investigated [73].

**Fig. 3:**
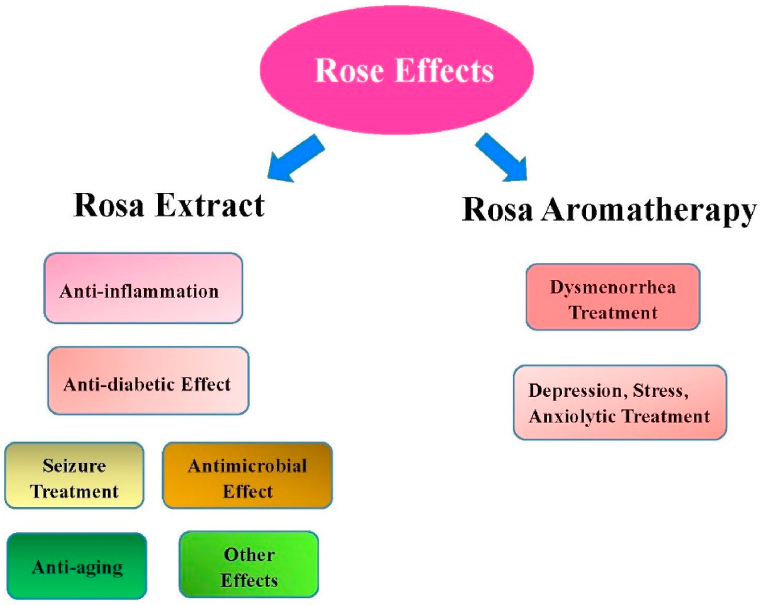
Medicinal uses of Roses.

The effects of rose discussed in this paper are summarized in Fig. 3.

## 4 Polymer and material application

The advantage of biopolymers being incinerated without emitting toxic residues is an important benefit associated with the use of biopolymers [107]. Cellulose, collagen, pectin, and gelatin are among the natural polymers extensively employed in various biomedical applications, including pharmaceuticals, tissue regeneration scaffolds, drug delivery agents, and more [38,108,109]. In this section, the material applications of roses are reviewed.

### 4.1 Biopolymer

#### 4.1.1 Biomimetic materials

**Fig. 4:**
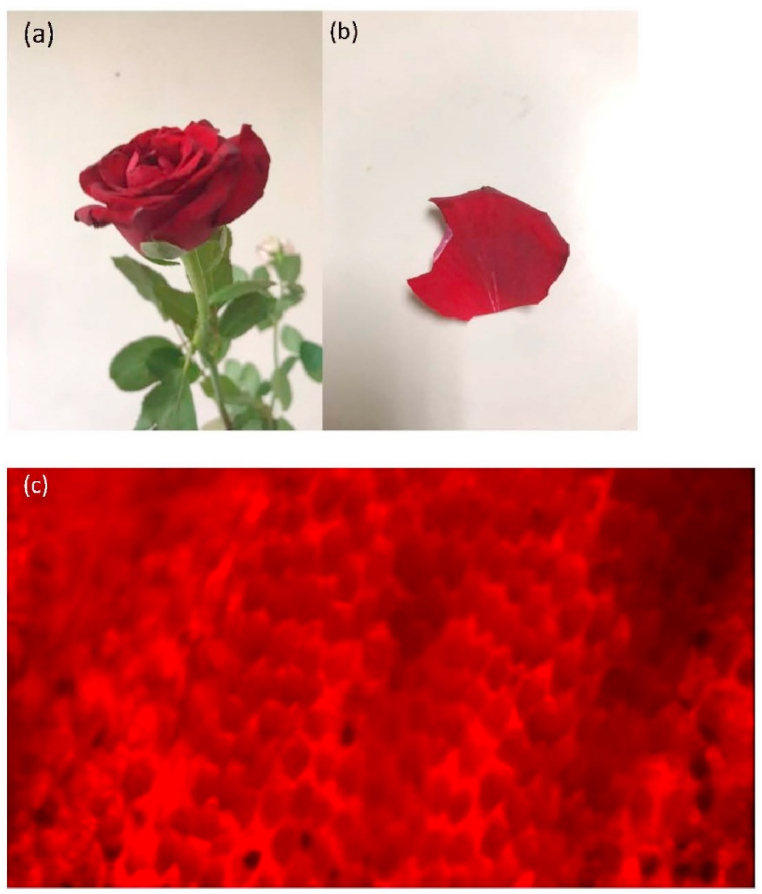
(a) Rose flower; (b) one petal; (c) the surface of the rose petal under 400× magnification.

Plant surfaces have evolved a variety of special functions such as water-repellent properties. The flower petals of many plants are superhydrophobic, but water droplets do not roll off when the surfaces are tilted [110]. These functions could provide an important source of inspiration for the design of biomimetic materials. The surface of the rose petal has a hierarchical structure consisting of microscale papillae, and each papilla has nanofolds. Fig. 4 (a)-(b) shows a rose and displays a rose petal, and Fig. 4(c) illustrates the nanofold structure of the petal. This feature makes rose petals have high water contact angles as well as droplet adhesion with antagonistic wetting properties. Extreme roughness and chemical variability are the basis for the rose petal effect [111]. The rose petal pattern, replicated using a nano-imprint lithography process, was formed on a glass substrate, and the surface of the glass substrate exhibited a high adhesive force and superhydrophobicity [112]. Inspired by lotus leaf and rose-petal, a superhydrophobic surface was created with the ability to effectively control its water adhesion force. This surface demonstrated the capability of transitioning between self-cleaning and water-capturing modes through multiple switching events [113].

The hierarchical structures of rose petals make roses excellent biomimetic∕bioinspired materials. Through the imitation of the hierarchical micro/nanostructures present on the surface of rose petals, a hydrophobic polymer film was manufactured [114]. The surface of the polyurethane acrylate film was replicated with rose petal structures using UV-based nanoimprint lithography. The introduction of a rose petal mimetic structure resulted in an inherently hydrophilic material exhibiting a high degree of hydrophobicity. The ultra-sensitive capacitive pressure sensors, successfully mimicking rose petals, exhibited high sensitivity and fast response times, which hold promise for soft electronics and potential applications in electronic skin (e-skin) [115].

#### 4.1.2 Organic electronic materials

Rose-based biopolymers have multiple applications. Organic electronic materials are organic molecules or polymers that show desirable electronic properties such as conductivity. Organic electronics research primarily focused on flexible and inexpensive versions of traditional semiconductor technologies [116]. Organic bioelectronic platforms known as electronic plants enable electronic interactions with plant systems. Roses and other plants can serve as organic electronic materials capable of conducting and processing both electronic and ionic signals. Rosa floribunda was used as a model plant in the first example of an electronic function added to a plant used [117]. To prepare the rose stem, the lower section was trimmed and the fresh cross-section was submerged in an aqueous solution of Poly(3,4-ethylenedioxythiophene)-sulfonate hydrogen (PEDOT-S:H). This allowed the uptake of the PEDOT-S:H solution into the xylem vascular channel. As a result, the PEDOT-S:H formed uniformly structured hydrogel wires, extending extensively within the tubular xylem channel. The xylem, leaves, veins, and signals of Rosa were used as foundational elements and functional components of the circuit, resulting in the successful establishment of the circuit's four essential constituents. However, in the first plant integrated electronics example, the devices and circuits could only be manufactured in localized regions of the plant. In another rose example, in deionized water, a water-soluble conjugated oligomer known as the sodium salt of bis[3,4-ethylenedioxythiophene]3thiophene butyric acid (ETE-S) was dissolved. Subsequently, a fresh cross-section of a rose cutting was submerged in this solution. The ETE-S compound effectively traversed the complete xylem system of the rose cutting, undergoing polymerization to generate coatings and wires. The study conclusively revealed that the synthesis of this conjugated oligomer facilitated the formation of extended oligomers and polymers throughout all regions of the xylem vascular tissue in the Rosa floribunda cutting, resulting in the creation of conducting wires spanning considerable distances [118]. These results show the potential of roses in developing electronic plant products. Since many exciting possibilities have raised from E-Plants technology, a fundamental understanding of the self-assembly/organization mechanisms and in vivo chemistry of materials can lead to the rational design of materials and systems, leading to advanced technologies [119].

#### 4.1.3 Electronic skin

The human skin possesses various characteristics, including elasticity, the ability to self-heal, and the capability to sense touch. E-skin is a device designed to replicate the properties of human skin while incorporating extra functionalities [120]. E-skin could help detect early signs of COVID-19 in frontline health workers, as well as monitor patients with COVID-19 [121]. Flexible e-skins have potential applications in wearable electronics and biomedicine. E-skins based on natural materials have found diverse applications, including touch sensing, motion monitoring, gas flow detection, and spatial pressure distribution. Dried rose petals and leaves have been used as the dielectric layer in capacitive-type e-skins [122]. This work might offer a general strategy for making plant-based flexible e-skins. The device demonstrated a wide operating pressure range, spanning from 0.6 Pa to 115 kPa, and exhibited exceptional stability during 5000 cycles of pressing or bending. These findings potentially presented a universal approach for developing flexible e-skins using plant-based materials. Elastomeric petals replicated from natural rose petals could be multifunctional substrates for stretchable and printable electronics [123]. In comparison to conventional flat polydimethylsiloxane substrates, elastomeric petals demonstrated an efficient ability to inhibit the spread of microcracks in the conducting layer deposited on top. The geometrical surfaces of rose petals were replicated and used to fabricate high-performance electronic skin [124].

#### 4.1.4 Solar cell application

Solar cells are useful devices in wide applications that can convert the energy of light into electricity by the photovoltaic effect. It is important to design solar cells that can harvest energy with a high level of efficiency. The hierarchical structures of Rosa ‘El Toro’ petals were employed as light-harvesting elements to optimize organic solar cells [125]. Their broadband and omnidirectional anti-reflection properties, as well as their light-harvesting abilities, were experimentally analyzed. Improvements in power conversion efficiency were reported after integrating these replicas onto optimized state-of-the-art organic solar cells. Nanocrystalline TiO2 DSSCs were developed using red and table rose extracts as natural sensitizers, containing anthocyanin pigments with hydroxyl/carboxylic groups [126]. These eco-friendly, cost-effective solar cells show promise.

#### 4.1.5 Graphene application

The Rose petal effect can be applied to the popular material graphene. A particular type of surface property with high contact angle and high drop adhesion is known as the Rose petal effect [111]. This effect is a result of the combination of microscale and nanoscale structures on the surface of the material, which creates a surface that is highly water-repellent, or hydrophobic. Researchers were attempting to replicate the water-repellent properties of rose petals by creating specific surface structures using graphene and other materials to enhance adhesion or wettability. To achieve super-wettability, a hierarchical structure resembling flower petals was designed and created by growing few-layer graphene nanosheets on silicon nanocone arrays [127]. $Co_{3}O_{4}$ was a promising candidate as an anode material for lithium-ion batteries. Graphene-embedded $Co_{3}O_{4}$ rose spheres were effectively integrated into a graphene structure. The resulting graphene-embedded $Co_{3}O_{4}$ rose spheres demonstrated a notable reversible capacity, favorable cyclic performance, and exceptional rate capability [128].

Graphene aerogel (GA) has demonstrated great potential for energy storage, environmental remediation, and high-performance sensors. A sandwich-like cell wall with a biomimetic rose-petal-like surface was constructed by the incorporation of renewable natural rubber latex (NRL) particles into the cell walls of GAs [129]. The application of this bionic design, GA/NRL, has been demonstrated for efficiently collecting water droplets from humid air and enhancing its solar-thermal harvesting capacity.

### 4.2 Oil extraction

**Fig. 5:**
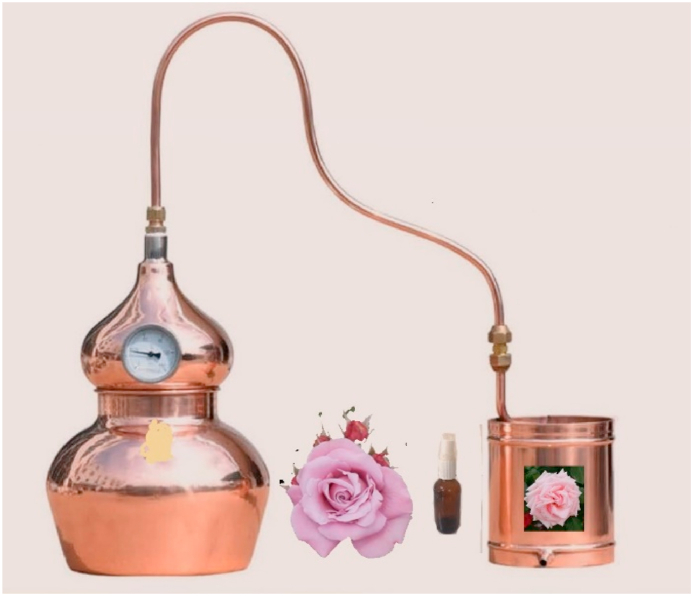
The rose extract can be homemade by steam distillation with simple equipment.

Rose essential oil is an herbal oil extracted from Rosa damascene that has been started since the 7th century A.D [130]. The main producers of rose essential oil in the world were Bulgaria, Turkey, and Morocco [131]. It can be used as a flavor additive in the food industry and an ingredient in the cosmetics industry. There are several main methods of extracting essential oil including steam distillation, solvent extraction, and supercritical CO2 extraction [80,132,133]. The production of rose oil from Rosa damascene traditionally involves the use of water steam distillation, a classical method [134]. During steam distillation, the plant material is exposed to dry steam, causing the volatilization of steam volatile compounds. These compounds are then condensed and collected in a receiver for further use. More advanced extraction methods or the comparison of the extraction method have been discussed in the literature. Four methods including ultrasound-assisted extraction, reflux extraction, Soxhlet extraction, and marinated extraction for the extraction of anthocyanins from red rose petals were investigated and compared [135]. Another study also compared four extraction methods including Soxhlet extraction, ultrasound-assisted extraction, and microwave-assisted extraction, as well as supercritical CO2 extraction [136]. Although there are other extraction methods, steam distillation has been commonly used for essential oil extraction. The rose extract can be homemade by steam distillation with simple equipment (Fig. 5). Rose is readily available and inexpensive. Therefore, rose is highly recommended for various purposes.

## 5 Conclusions

Rose symbolizes romance, love, beauty, and courage. It has been regarded as the king or queen of flowers. The benefits of its multiple functions outweigh most other flowers. The beauty of rose can comfort people; the fragrance of rose can calm stressed-out body and mind; the ingredients of rose can be a good source of nutrients and medicinal herbs; the hierarchical structures of rose petals have anti-reflection and light-harvesting abilities, which have the potential to be materials for various electronic products.

In this paper, the beneficial effects of rose extracts and the various applications of rose-based biopolymers are reviewed. The medical effects discussed in this paper can be categorized into two parts. The first part covers rose aromatherapy, including its effectiveness in dysmenorrhea treatment, as well as its potential for alleviating depression, stress, and anxiety. The second part focuses on the therapeutic uses of rose extracts, encompassing their anti-inflammatory and anti-diabetic effects, their role in seizure treatment, antimicrobial properties, anti-aging and skin repair benefits, and other effects.

The applications of rose-based biopolymers discussed in this paper encompass a range of areas, including biomimetic materials, organic electronic materials, electronic skin, solar cell applications, and graphene utilization.

During the COVID-19 pandemic, the shortage of medical materials has been becoming a global issue. Exploring natural biomaterials as viable substitutes has emerged as a promising alternative. Roses with so many benefits definitely deserve more exploration and promotion.

## Funding

This research was funded by the Ministry of Science and Technology 111-2118-*M*-A49-001-MY2, Taiwan.

## Data availability statement

No data was used for the research described in the article.

## CRediT authorship contribution statement

**Hsiuying Wang:** Writing – review & editing, Writing – original draft, Resources, Investigation, Funding acquisition, Conceptualization.

## Declaration of competing interest

The authors declare that they have no known competing financial interests or personal relationships that could have appeared to influence the work reported in this paper.
